# Comparing Indirect Effects in Different Groups in Single-Group and Multi-Group Structural Equation Models

**DOI:** 10.3389/fpsyg.2017.00747

**Published:** 2017-05-11

**Authors:** Ehri Ryu, Jeewon Cheong

**Affiliations:** ^1^Psychology, Boston CollegeChestnut Hill, MA, USA; ^2^Health Education and Behavior, University of FloridaGainesville, FL, USA

**Keywords:** moderated mediation, moderated indirect effect, group difference in mediation, multi-group analysis, simple indirect effect

## Abstract

In this article, we evaluated the performance of statistical methods in single-group and multi-group analysis approaches for testing group difference in indirect effects and for testing simple indirect effects in each group. We also investigated whether the performance of the methods in the single-group approach was affected when the assumption of equal variance was not satisfied. The assumption was critical for the performance of the two methods in the single-group analysis: the method using a product term for testing the group difference in a single path coefficient, and the Wald test for testing the group difference in the indirect effect. Bootstrap confidence intervals in the single-group approach and all methods in the multi-group approach were not affected by the violation of the assumption. We compared the performance of the methods and provided recommendations.

## Introduction

In mediation analysis, it is a standard practice to conduct a formal statistical test on mediation effects in addition to testing each of the individual parameters that constitutes the mediation effect. Over the past few decades, statistical methods have been developed to achieve valid statistical inferences about mediation effects. The sampling distribution of a mediation effect is complicated because the mediation effect is quantified by a product of at least two parameters. For this reason, numerous studies have proposed and recommended methods that do not rely on distributional assumption (e.g., bootstrapping) for testing mediation effects (e.g., Bollen and Stine, 1990; Shrout and Bolger, 2002; MacKinnon et al., 2004; Preacher and Hayes, 2004).

It is often a question of interest whether a mediation effect is the same across different groups of individuals or under different conditions, in other words, whether a mediation effect is moderated by another variable (called a moderator) that indicates the group membership or different conditions. For example, Levant et al. (2015) found that the mediation effect of endorsement of masculinity ideology on sleep disturbance symptoms via energy drink use was significantly different between white and racial minority groups. Schnitzspahn et al. (2014) found that time monitoring mediated the effect of mood on prospective memory in young adults, but not in old adults. Gelfand et al. (2013) showed that the effect of cultural difference (US vs. Taiwan) on the optimality of negotiation outcome is mediated by harmony norm when negotiating as a team but not when negotiating as solos. In these studies, the mediation effect was moderated by a categorical moderator (e.g., racial group, age group, experimental condition). With a categorical moderator, the moderated mediation effect concerns the difference in the indirect effect between groups. Treating a moderator categorical is appropriate when the moderator is truly categorical, but it is not appropriate to create groups based on arbitrary categorization of a continuous moderator (Maxwell and Delaney, 1993; MacCallum et al., 2002; Edwards and Lambert, 2007; Rucker et al., 2015).

Structural equation modeling (SEM) is a popular choice for many researchers to test a mediation model and to conduct a formal test on mediation effects. In SEM, the mediation effect can be specified as an indirect effect (Alwin and Hauser, 1975; Bollen, 1987) such as “the indirect effect of an independent variable (X) on a dependent variable (Y) via a mediator (M)” in which X affects M, which in turn affects Y. For incorporating a categorical moderator, there are two approaches in SEM: single-group and multi-group analysis. In the single-group analysis approach, the categorical moderator is represented by a variable, or a set of variables, in the model. On the other hand, the multi-group analysis approach uses the categorical moderator to separate the observations into groups at each level of the moderator, and the moderator does not appear in the model as a variable.

In this article, we present the single-group and multi-group analysis approaches to comparing indirect effects between groups, and introduce statistical methods in each approach for testing the group difference in the indirect effect and for testing the simple indirect effect in each group. Then we present a simulation study to compare the performance of the methods. In particular, we examine how robust the methods in single-group analysis approach are when the assumption of homogeneity of variance is not satisfied (the assumption is described in a later section).

## Group difference in indirect effect and simple indirect effect in each group

**Figure 1:**
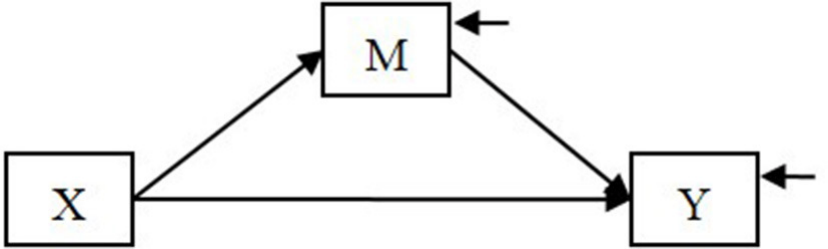
**A mediation model**.

We use the following example throughout this article. Suppose that we hypothesize a mediation model in which the effect of an independent variable X on a dependent variable Y is mediated by a mediator M (Figure 1).

We also hypothesize that the X to M relationship is not the same in two groups of individuals (e.g., men and women). This model can be considered as a special case of the first *stage moderation model* in Edwards and Lambert (2007) and the *Model 2* in Preacher et al. (2007), in which the moderator is a categorical variable with two levels. When comparing the indirect effect between two groups, estimating and making statistical inferences about the following two effects are of interest. First, what is the estimated difference in the indirect effect between the groups? Second, what is the estimated indirect effect in each group (i.e., simple indirect effect)?

**Figure 2:**
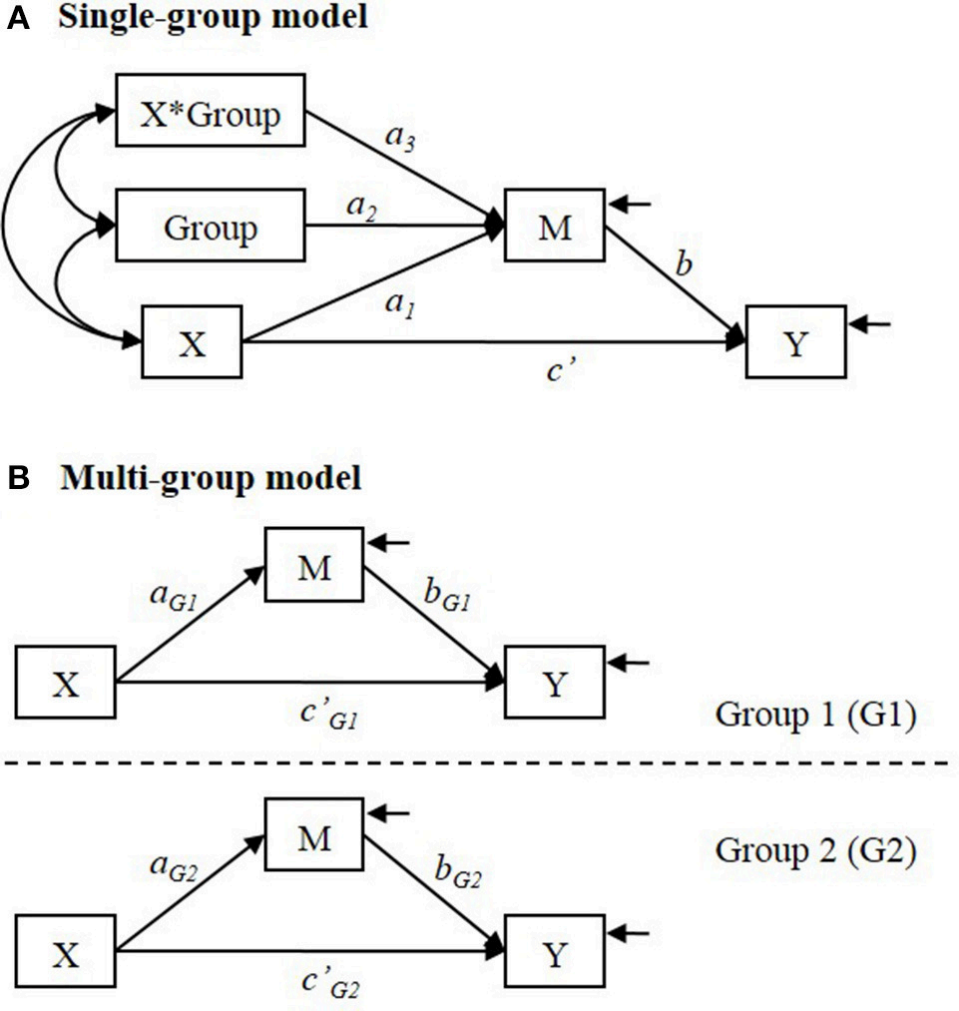
**(A)** Single-group and **(B)** multi-group analysis models for testing group difference in the indirect effect. In **(A)** single-group model, Group is a categorical variable that indicates distinctive group membership.

In the single-group analysis, a (set of) categorical variable indicating the group membership is used as a covariate in the model and an interaction term of X with the group membership (Group) is included to test the difference in the X to M relationship between groups (See Figure 2A).

The interpretation of the parameters depends on how the group membership is coded. For example, when the group membership (Group) is dummy coded as 1 = Group 1 and 0 = Group 2, *a*_1_ = simple effect of X on M in Group 2; *a*_2_ = group difference in conditional mean of M for those whose level of X is at zero (i.e., conditional mean of M in Group 1—conditional mean of M in Group 2); *a*_3_ = difference in simple effect of X on M between groups (i.e., simple effect of X on M in Group 1—simple effect of X on M in Group 2). If *a*_3_ ≠ 0, it means that the relationship between X and M is not the same between groups.

When the relationship between X and M differs between groups, the indirect effect of X on Y via M is conditional on the group membership, because the indirect effect consists of X to M relationship and M to Y relationship. In the model shown in Figure 2A, an estimate of the indirect effect of X on Y via M is obtained by $\left[{â}_{1}+{â}_{3}\left(Group\right)\right]\hat{b}$ (Preacher et al., 2007). So the simple indirect effect (i.e., the conditional indirect effect) estimate is $\left[{â}_{1}+{â}_{3}\left(1\right)\right]\hat{b}$ = $\left({â}_{1}+{â}_{3}\right)\hat{b}$ in Group 1 (coded 1), and $\left[{â}_{1}+{â}_{3}\left(0\right)\right]\hat{b}$ = ${â}_{1}\hat{b}$ in Group 2 (coded 0). The estimated group difference in the indirect effect is $\left[\left({â}_{1}+{â}_{3}\right)\hat{b}\right]-{â}_{1}\hat{b}$ = ${â}_{3}\hat{b}$ (Hayes, 2015).

In multi-group analysis, group membership is not used as a predictor variable in the model. Instead, a set of hypothesized models (e.g., a set of two models if there are two distinctive groups) are specified and estimated simultaneously (See Figure 2B). The group difference in the simple effect of X on M (that is estimated by *a*_3_ in the single-group analysis) is estimated by (â_*G*1_−â_*G*2_). The simple indirect effect is estimated by ${â}_{G1}{\hat{b}}_{G1}$ and ${â}_{G2}{\hat{b}}_{G2}$ in Group 1 and in Group 2, respectively. The estimated difference in the indirect effect is $\left({â}_{G1}{\hat{b}}_{G1}-{â}_{G2}{\hat{b}}_{G2}\right)$.

## Statistical inferences

There are numerous methods for making statistical inferences about the simple indirect effects and inferences about the group difference in the indirect effect. The methods can be categorized into the following branches: (1) normal-theory standard error, (2) bootstrapping methods, (3) Monte Carlo method, (4) likelihood ratio (LR) test, (5) Wald test^1^. Table 1 summarizes the methods and shows the abbreviation to refer to each method. In the abbreviation, the superscripts “S” and “M” indicate the single-group and multi-group approaches, respectively. The subscripts indicate which effect is tested by the method, e.g., “diff” means the group difference in the indirect effect, “ind” means the simple indirect effect in in each group.

**Table 1:** **Methods for testing group difference in a path, group difference in the indirect effect, and simple indirect effect in each group**.

	**Abbreviation**	**Description**
**SINGLE-GROUP ANALYSIS**		
Group difference in *a* path	$z_{a3}^{S}$	*z* = â_3_/*se*_*a*3_
Group difference in the indirect effect	$W_{diff}^{S}$	Wald test for *a*_3_*b* = 0
Simple indirect effect in each group	$PC_{ind}^{S}$	Percentile bootstrap CI for the simple indirect effect in each group
	$BC_{ind}^{S}$	Bias-corrected bootstrap CI for the simple indirect effect in each group
**MULTI-GROUP ANALYSIS**		
Group difference in *a* path	$LR_{a}^{M}$	Likelihood ratio test for *a*_*G*1_ = *a*_*G*2_
Group difference in the indirect effect	$LR_{diff}^{M}$	Likelihood ratio test for *a*_*G*1_*b*_*G*1_ = *a*_*G*2_*b*_*G*2_
	$W_{diff}^{M}$	Wald test for *a*_*G*1_*b*_*G*1_ = *a*_*G*2_*b*_*G*2_
	$PC_{diff}^{M}$	Percentile bootstrap CI for the group difference in the indirect effect
	$BC_{diff}^{M}$	Bias-corrected bootstrap CI for the group difference in the indirect effect
	$MC_{diff}^{M}$	Monte Carlo CI for the group difference in the indirect effect
Simple indirect effect in each group	$PC_{ind}^{M}$	Percentile bootstrap CI for the simple indirect effect in each group
	$BC_{ind}^{M}$	Bias-corrected bootstrap CI for the simple indirect effect in each group
	$MC_{ind}^{M}$	Monte Carlo confidence interval for the simple indirect effect in each group

*The superscripts “S” and “M” indicate the single-group and multi-group approaches, respectively. The subscript “ind” indicates the simple indirect effect in each group; the subscript “diff” indicates the group difference in the indirect effect. CI, confidence interval. We used 95% confidence for all interval estimates*.

### Normal-theory standard error

The normal-theory standard error method is based on the assumption that the sampling distribution of the estimate follows a normal distribution. In testing an indirect effect, it is well-known that the normality assumption is not appropriate to represent the sampling distribution of the indirect effect, and the normal-theory based method do not perform well in testing the indirect effect (e.g., MacKinnon et al., 2002; Shrout and Bolger, 2002; MacKinnon et al., 2004; Preacher and Selig, 2012). In moderated mediation models, Preacher et al. (2007) has advocated the bootstrapping methods over the normal standard error methods for testing the simple indirect effect.

### Bootstrapping methods

The bootstrapping methods can provide interval estimates without relying on a distribution assumption. For this reason, the bootstrapping methods have been recommended for testing indirect effects in previous studies (e.g., MacKinnon et al., 2004; Preacher and Hayes, 2004). The bootstrapping methods can be applied for obtaining interval estimates for any effect of interest, e.g., simple indirect effect in Group 1, simple indirect effect in Group 2, group difference in the indirect effect. In bootstrapping methods, a large number of bootstrap samples (e.g., 1,000 bootstrap samples), whose sizes are the same as the original sample size, are drawn from the original sample with replacement. An estimate is obtained in each bootstrap sample. An empirical sampling distribution is constructed using the set of 1,000 bootstrap estimates. From the bootstrap sampling distribution, percentile bootstrap confidence intervals ([100 ^*^ (1−α)]%) can be computed by the (α/2) and (1−α/2) percentiles. Bias-corrected bootstrap confidence intervals can be computed with the percentiles adjusted based on the proportion of bootstrap estimates lower than the original sample estimate (see MacKinnon et al., 2004).

In the single-group analysis, the estimate of the simple indirect effect in each group is computed by $\left({â}_{1}^{*}+{â}_{3}^{*}\right){\hat{b}}^{*}$ in Group 1 (coded 1), and ${â}_{1}^{*}{\hat{b}}^{*}$ in Group 2 (coded 0) in each bootstrap sample. The superscript ^*^ denotes that the estimates are obtained in bootstrap samples. In each group, the percentile ($PC_{ind}^{S}$ in Table 1) and the bias-corrected ($BC_{ind}^{S}$) bootstrap confidence intervals for the simple indirect effect are computed from the bootstrap sampling distribution [i.e., the distribution of $\left({â}_{1}^{*}+{â}_{3}^{*}\right){\hat{b}}^{*}$ for Group 1; and the distribution of ${â}_{1}^{*}{\hat{b}}^{*}$ for Group 2] as described above.

In the multi-group analysis, the estimate of the simple indirect effect is computed by ${â}_{G1}^{*}{\hat{b}}_{G1}^{*}$ in Group 1 and ${â}_{G2}^{*}{\hat{b}}_{G2}^{*}$ in Group 2. The percentile ($PC_{ind}^{M}$) and the bias-corrected ($BC_{ind}^{M}$) bootstrap confidence intervals for the simple indirect effect are obtained from the distribution of ${â}_{G1}^{*}{\hat{b}}_{G1}^{*}$ and the distribution of ${â}_{G2}^{*}{\hat{b}}_{G2}^{*}$, in Group 1 and Group 2, respectively. The percentile ($PC_{diff}^{M}$) and the bias-corrected ($BC_{diff}^{M}$) bootstrap confidence intervals for the group difference in the indirect effect are obtained from the bootstrap sampling distribution of $\left({â}_{G1}^{*}{\hat{b}}_{G1}^{*}-{â}_{G2}^{*}{\hat{b}}_{G2}^{*}\right)$.

### Monte carlo method

The Monte Carlo method provides a statistical test or an interval estimate of an effect by generating parameter values with a distributional assumption (e.g., multivariate normal). For testing the group difference in the indirect effect in the multi-group analysis model, the parameter estimates and standard errors are used to specify a joint sampling distribution of the parameter estimates from which the parameter values are generated for a large number of replications, e.g., 1,000 (Preacher and Selig, 2012; Ryu, 2015), such that the joint distribution of the four parameters *a*_*G*__1_, *b*_*G*__1_, *a*_*G*__2_, and *b*_*G*__2_ is a multivariate normal distribution shown below.

(1) $$\left[\begin{array}{c} a_{G1}\\ b_{G1}\\ a_{G2}\\ b_{G2} \end{array}\right]\sim MVN\left(\left[\begin{array}{c} {\hat{a}}_{G1}\\ {\hat{b}}_{G1}\\ {\hat{a}}_{G2}\\ {\hat{b}}_{G2} \end{array}\right],\left[\begin{array}{cccc} {\hat{\sigma }}_{a_{G1}}^{2} & & & \\ 0 & {\hat{\sigma }}_{b_{G1}}^{2} & & \\ 0 & 0 & {\hat{\sigma }}_{a_{G2}}^{2} & \\ 0 & 0 & 0 & {\hat{\sigma }}_{b_{G2}}^{2} \end{array}\right]\right)$$

where â_*G*1_, ${\hat{b}}_{G1}$, â_*G*2_, and ${\hat{b}}_{G2}$ are the estimates in the original sample, and ${\hat{\sigma }}_{a_{G1}}$, ${\hat{\sigma }}_{b_{G1}}$, ${\hat{\sigma }}_{a_{G2}}$, and ${\hat{\sigma }}_{b_{G2}}$ are the estimated standard errors in the original sample. The parameters in Group 1 (*a*_*G*1_, *b*_*G*1_) are independent of the parameters in Group 2 (*a*_*G*2_, *b*_*G*2_) because Group 1 and Group 2 are independent as long as the assumption of independent observations is valid. In mediation model, the covariance between *a* and *b* paths are often replaced with zero (Preacher and Selig, 2012). So the covariance between *a* and *b* paths is zero in each group (${\hat{\sigma }}_{b_{G1},a_{G1}}$ = 0; ${\hat{\sigma }}_{b_{G2},a_{G2}}$ = 0). For a large number of replications, parameter values ${â}_{G1}^{+}$, ${\hat{b}}_{G1}^{+}$, ${â}_{G2}^{+}$, and ${\hat{b}}_{G2}^{+}$ are generated from the multivariate normal distribution shown in (1). The superscript + denotes the parameter values generated by Monte Carlo method. In each replication, the simple indirect effect estimate is computed by ${â}_{G1}^{+}{\hat{b}}_{G1}^{+}$ in Group 1 and by ${â}_{G2}^{+}{\hat{b}}_{G2}^{+}$ in Group 2. The group difference in the indirect effect is computed by $\left({â}_{G1}^{+}{\hat{b}}_{G1}^{+}-{â}_{G2}^{+}{\hat{b}}_{G2}^{+}\right)$. The Monte Carlo confidence intervals ([100 ^*^ (1−α)]%) are obtained by the (α/2) and (1−α/2) percentiles in the set of generated values. For the simple indirect effect in Group 1, the Monte Carlo confidence intervals ($MC_{ind}^{M}$) are computed using the set of ${â}_{G1}^{+}{\hat{b}}_{G1}^{+}$ values, and using the set of ${â}_{G2}^{+}{\hat{b}}_{G2}^{+}$ values in each group, respectively. The Monte Carlo confidence interval for the group difference in the indirect effect ($MC_{diff}^{M}$) is obtained using the set of $\left({â}_{G1}^{+}{\hat{b}}_{G1}^{+}-{â}_{G2}^{+}{\hat{b}}_{G2}^{+}\right)$ values. The Monte Carlo method is less computer-intensive and less time-consuming than the bootstrapping method.

### Likelihood ratio test

The likelihood ratio (LR) test and the Wald test can be used to test a (set of) constraint. The LR test (Bentler and Bonett, 1980; Bollen, 1989) is obtained by estimating two nested models with (M_1_) and without (M_0_) the constraints. The LR test results in a chi-square statistic with the degrees of freedom (df) equal to the difference in the number of freely estimated parameters in the two models.

(2) $$\chi^{2}=-2log\left[\frac{L\left(M_{1}\right)}{L\left(M_{0}\right)}\right]=\left\{-2log\left[L\left(M_{1}\right)\right]\right\}-\{-2log\left[L\left(M_{0}\right)\right]\}$$

where *L*(*M*_*k*_) = likelihood of model *k*. The LR test can be used to test the group difference in the “X → M” relationship in the multi-group analysis model, by comparing two models with and without the constraint *a*_*G*1_ = *a*_*G*2_, with df = 1 ($LR_{a}^{M}$). Likewise, the LR test can be used to test the group difference in the indirect effect by comparing two models with and without the constraint *a*_*G*1_*b*_*G*1_ = *a*_*G*2_*b*_*G*2_, with df = 1 ($LR_{diff}^{M}$).

### Wald test

The Wald test (Wald, 1943; Bollen, 1989) evaluates a constraint in a model in which the constraint is not imposed. For testing group difference in the indirect effect, the constraint *a*_3_*b* = 0 is tested in the single-group analysis ($W_{diff}^{S}$). The Wald statistic (with df = 1) is obtained by

(3) $$W={\hat{\theta }}_{1}^{2}/avar\left({\hat{\theta }}_{1}\right)$$

Where θ_1_ = *a*_3_*b* and $avar\left({\hat{\theta }}_{1}\right)$ = estimated asymptotic variance of ${\hat{\theta }}_{1}$, i.e., estimated asymptotic variance of ${â}_{3}\hat{b}$. Likewise, for testing group difference in the indirect effect in the multi-group model, the constraint *a*_*G*1_*b*_*G*1_ = *a*_*G*2_*b*_*G*2_ is tested ($W_{diff}^{M}$). The Wald statistic (df = 1) is obtained by (3) with θ_1_ = *a*_*G*1_*b*_*G*1_−*a*_*G*2_*b*_*G*2_ in the multi-group model.

A previous simulation study (Ryu, 2015) compared the performance of different methods for testing group difference in the indirect effect in multi-group analysis. In the previous study, the LR test performed well in terms of Type I error rate and statistical power. The percentile bootstrap confidence intervals for the group difference in indirect effect showed coverage rates that are close to the nominal level. The bias-corrected bootstrap confidence intervals were more powerful than the percentile bootstrap confidence intervals but the bias-corrected bootstrap confidence intervals showed inflated Type I error rates.

## Single-group and multi-group approaches

The multi-group analysis model shown in Figure 2B is less restrictive the single-group analysis model shown in Figure 2A. In the single-group model shown in Figure 2A, *b* and *c*′ paths are assumed to be equal between groups, whereas *b* and *c*′ paths are allowed to differ between groups in the multi-group model, unless additional equality constraints are imposed. It is possible to specify a single-group model that allow *b* or *c*′ paths to differ between groups. In order to allow these parameters to differ between groups in the single-group model, additional parameters need to be estimated or additional interaction terms need to be added. If the model shown in Figure 2A is modified by specifying the path coefficients “Group → Y” and “X^*^Group → Y” to be freely estimated, that will allow *c*′ to differ between groups. In order to allow *b* to differ between groups, the model needs an additional variable “M^*^Group” and the path coefficients “Group → Y” and “M^*^Group → Y” need to be freely estimated. The multi-group model can be simplified by imposing equality constraints ${\hat{b}}_{G1}={\hat{b}}_{G2}$ and / or ĉ′_G1_ = ĉ′_G2_.

In the single-group model, the variance and covariance parameters are assumed to be equal as well, whereas in the multi-group model those parameters are not restricted to be the same between groups unless additional equality constraints are imposed. Specifically, in the single-group analysis model (as shown in Figure 2A) the residual variances of M and Y are assumed to be equal in both groups. The equal variance assumption in the single-group analysis is one of the standard assumptions in general linear models. The assumption is that the conditional variance of the dependent variable is homogeneous at all levels of the independent variables. For example, in regression analysis, the conditional variance of the dependent variable is assumed to be equal at all levels of the predictor variable. In between-subject analysis of variance or in *t*-test to compare two independent means, the within-group variance is assumed to be equal across all groups. It is well-known that the empirical Type I error rate can be different from the nominal level when the equal variance assumption is violated (e.g., Box, 1954; Glass et al., 1972; Dretzke et al., 1982; Aguinis and Pierce, 1998).

The purpose of this study is to introduce the single-group and multi-group approaches in SEM to comparing indirect effects between groups, and to empirically evaluate the performance of the statistical methods. Specifically, we aim to empirically evaluate how well the statistical methods (summarized in Table 1) perform for three questions in the moderated mediation model: (i) comparing the *a* path (X → M) between groups, (ii) comparing the indirect effect between groups, (iii) testing simple indirect effect in each group. The methods we considered are summarized in Table 1. We also evaluate how robust the methods in the single-group analysis are when the assumption of equal variances does not hold between groups. We expected that the performance of the methods in multi-group analysis would not be affected by the violation of the assumption of equal variances, because the multi-group analysis model does not rely on the assumption. In the single-group analysis, we expected that the performance of the $z_{a3}^{S}$ and $W_{diff}^{S}$ methods would be affected by the violation of the equal variance assumption, and that the confidence intervals produced by the bootstrapping methods ($PC_{ind}^{S}$, $BC_{ind}^{S}$) would not be affected by the violation of the assumption. The estimates are expected to be unbiased regardless of the equal variance assumption violated. The bootstrap sampling distribution is constructed using the estimates in bootstrap samples. Therefore, as long as the violation of the equal variance assumption does not affect the unbiasedness of the estimates, the performance of the bootstrap confidence intervals is not expected to be affected by the violation of the assumption.

## Simulation

We used the mediation model shown in Figure 2B as the population model. There were two distinctive groups (denoted by G1 and G2). We considered a total of 63 conditions: 21 populations × 3 sample sizes.

As shown in Table 2, the 21 populations were created by combinations of three sets of parameter values for structural paths (Populations I, II, and III) and seven sets of parameter values for residual variances (Populations -0, -M1, -M2, -M3, -Y1, -Y2, -Y3). In Population I, there was no group difference in the indirect effect (*a*_*G*1_*b*_*G*1_ = 0.165; *a*_*G*2_*b*_*G*2_ = 0.165). In Population II, there was no indirect effect in G1; there was a small indirect effect in G2 (*a*_*G*1_*b*_*G*1_ = 0.000; *a*_*G*2_*b*_*G*2_ = 0.055); the group difference in the indirect effect was (*a*_*G*1_*b*_*G*1_−*a*_*G*2_*b*_*G*2_) = −0.055. In Population III, there was no indirect effect in G1; there was a large indirect effect in G2 (*a*_*G*1_*b*_*G*1_ = 0.000; *a*_*G*2_*b*_*G*2_ = 0.165); the group difference in the indirect effect was –0.165. The direct effect of X on Y was set to zero (i.e., ĉ′_*G*1_ = ĉ′_*G*2_ = 0) in all populations. It has been shown in a previous simulation study (Ryu, 2015) that the population value of the direct effect had little influence on the performance of the five methods for testing the group difference in indirect effect. With each set of the parameter values for structural paths, there were seven patterns of residual variances of M and Y. In Population -0, the residual variances of M and Y were equal between the groups in the population. In Populations -M1, -M2, and -M3, the residual variance of M was smaller in G1. In Populations -Y1, -Y2, and -Y3, the residual variance of Y was smaller in G1. Note that the effect sizes varied depending on the residual variances. The proportions of explained variance in M and Y in the 21 populations are summarized in Table 3.

**Table 2:** **Parameter values for structural paths *a* and *b*, and for residual variances of M and Y in population**.

**Population**	**Parameter values**
**PARAMETER VALUES FOR STRUCTURAL PATHS**	
Population I	*a*_*G*1_ = 0.424, *b*_*G*1_ = 0.390; *a*_*G*2_ = 0.424, *b*_*G*2_ = 0.390
Population II	*a*_*G*1_ = 0.000, *b*_*G*1_ = 0.390; *a*_*G*2_ = 0.141, *b*_*G*2_ = 0.390
Population III	*a*_*G*1_ = 0.000, *b*_*G*1_ = 0.390; *a*_*G*2_ = 0.424, *b*_*G*2_ = 0.390
**PARAMETER VALUES FOR RESIDUAL VARIANCES**	
0	ψ_*M*(*G*1)_ = 1.0, ψ_*Y*(*G*1)_ = 1.0; ψ_*M*(*G*2)_ = 1.0, ψ_*Y*(*G*2)_ = 1.0
M1	ψ_*M*(*G*1)_ = 0.5, ψ_*Y*(*G*1)_ = 1.0; ψ_*M*(*G*2)_ = 1.0, ψ_*Y*(*G*2)_ = 1.0
M2	ψ_*M*(*G*1)_ = 0.5, ψ_*Y*(*G*1)_ = 1.0; ψ_*M*(*G*2)_ = 1.5, ψ_*Y*(*G*2)_ = 1.0
M3	ψ_*M*(*G*1)_ = 0.5, ψ_*Y*(*G*1)_ = 1.0; ψ_*M*(*G*2)_ = 2.0, ψ_*Y*(*G*2)_ = 1.0
Y1	ψ_*M*(*G*1)_ = 1.0, ψ_*Y*(*G*1)_ = 0.5; ψ_*M*(*G*2)_ = 1.0, ψ_*Y*(*G*2)_ = 1.0
Y2	ψ_*M*(*G*1)_ = 1.0, ψ_*Y*(*G*1)_ = 0.5; ψ_*M*(*G*2)_ = 1.0, ψ_*Y*(*G*2)_ = 1.5
Y3	ψ_*M*(*G*1)_ = 1.0, ψ_*Y*(*G*1)_ = 0.5; ψ_*M*(*G*2)_ = 1.0, ψ_*Y*(*G*2)_ = 2.0

*21 populations were created by 3 (structural paths) by 7 (residual variances) combinations, e.g., Population I–0, Population I–M1, …, Population III-Y3. The direct effects of X on Y ĉ′_G1_ = ĉ′_G2_ = 0 in all populations*.

**Table 3:** **Proportion of explained variance in M and Y in population**.

**Population**	**Group 1**		**Group 2**	
	**M**	**Y**	**M**	**Y**
I-0	0.152	0.152	0.152	0.152
I-M1	0.264	0.094	0.152	0.152
I-M2	0.264	0.094	0.107	0.204
I-M3	0.264	0.094	0.082	0.249
I-Y1	0.152	0.264	0.152	0.152
I-Y2	0.152	0.264	0.152	0.107
I-Y3	0.152	0.264	0.152	0.082
II-0	0.000	0.132	0.019	0.134
II-M1	0.000	0.071	0.019	0.134
II-M2	0.000	0.071	0.013	0.188
II-M3	0.000	0.071	0.010	0.235
II-Y1	0.000	0.233	0.019	0.134
II-Y2	0.000	0.233	0.019	0.094
II-Y3	0.000	0.233	0.019	0.072
III-0	0.000	0.132	0.152	0.152
III-M1	0.000	0.071	0.152	0.152
III-M2	0.000	0.071	0.107	0.204
III-M3	0.000	0.071	0.082	0.249
III-Y1	0.000	0.233	0.152	0.152
III-Y2	0.000	0.233	0.152	0.107
III-Y3	0.000	0.233	0.152	0.082

*See Table 2 for population parameter values*.

We considered three different sample sizes for each of the 21 populations. Sample size 1: *n*_*G*1_ = 150; *n*_*G*2_ = 150. Sample size 2: *n*_*G*1_ = 200; *n*_*G*2_ = 100. Sample size 3: *n*_*G*1_ = 100; *n*_*G*2_ = 200. With Sample size 2, the residual variances were smaller in the larger group. With Sample size 3, the residual variances were smaller in the smaller group. We used Mplus 7 for data generation and estimation (Muthén and Muthén, 1998–2012). We used SAS PROC IML for resampling of the data to create bootstrap samples. We conducted 1,000 replications in each condition.

We analyzed each of the generated data sets both in single-group analysis (0 = Group 1, 1 = Group 2) and in multi-group analysis to test the group difference in *a* path, the group difference in the indirect effect of X on Y via M, and the simple indirect effect in each group. We used the methods summarized in Table 1. We provide the sample syntax for data generation and analysis in the Appendix.

### Evaluation of methods

In order to check the data generation and estimation, we first examined the bias of the estimates. Bias was computed by (mean of estimates–true value in the population). Relative bias was computed by (bias/true value in the population) for the effects whose population values were not zero. In the single-group analysis, we compared the following estimates to their corresponding population values: individual path coefficients â_1_, â_3_, $\hat{b}$, the simple indirect effect in Group 1 ${â}_{1}\hat{b}$, and the simple indirect effect in Group 2 $\left({â}_{1}+{â}_{3}\right)\hat{b}$. In the multi-group analysis, we compared the following estimates to their corresponding population values: individual path coefficients â_*G*1_, ${\hat{b}}_{G1}$, â_*G*2_, ${\hat{b}}_{G2}$, the simple indirect effects in each group ${â}_{G1}{\hat{b}}_{G1}$, ${â}_{G2}{\hat{b}}_{G2}$, and the group difference in the indirect effect $\left({â}_{G1}{\hat{b}}_{G1}-{â}_{G2}{\hat{b}}_{G2}\right)$.

To evaluate the performance of the methods, we examined the rejection rates that can be interpreted as Type I error rate (when the effect was zero in population) or statistical power (when there was a non-zero effect in population) for each method. For the z test of *a*_3_ path ($z_{a3}^{S}$), LR test ($LR_{a}^{M}$, $LR_{diff}^{M}$), and Wald test ($W_{diff}^{S}$, $W_{diff}^{M}$), we used α = 0.05 criterion. For confidence intervals (95%), we computed the rejection rate by the proportion of replications in which the interval estimates did not include zero. We also examined coverage rates, width of confidence intervals, rate of left-side misses, rate of right-side misses, and ratio of left-side misses to right-side misses for interval estimates.

## Results

As expected, the estimates were unbiased in all populations with all sample sizes. In the single-group analysis, the bias ranged from 0.007 to −0.005, and the relative bias ranged from −0.038 to 0.007. The estimates obtained in the single-group analysis were unbiased regardless of whether the assumption of equal residual variances was satisfied. In the multi-group analysis, the bias ranged from −0.004 to 0.007, and the relative bias ranged from −0.011 to 0.051.

We present the simulation results in three sections: methods for testing the group difference in *a* path, methods for testing the group difference in the indirect effect, and methods for testing simple indirect effect in each group.

### Group difference in *a* path

Table 4 shows the empirical Type I error rates (nominal α = 0.05) of the methods for testing the group difference in *a* path in single-group ($z_{a3}^{S}$) and multi-group analysis ($LR_{a}^{M}$) in Population I.

**Table 4:** **Type I error rates of the methods for testing group difference in a path**.

			**Sample size**			
	***n*_*G*1_ = 150; *n*_*G*2_ = 150**		***n***_*****G***1**_ **=** **200;** ***n***_*****G***2**_ **=** **100**		***n***_*****G***1**_ **=** **100;** ***n***_*****G***2**_ **=** **200**	
**Population**	**$z_{a3}^{S}$**	**$LR_{a}^{M}$**	**$z_{a3}^{S}$**	**$LR_{a}^{M}$**	**$z_{a3}^{S}$**	**$LR_{a}^{M}$**
I-0	0.051	0.049	0.052	0.051	0.056	0.053
I-M1	0.055	0.052	**0.086**	0.056	0.031	0.053
I-M2	0.048	0.051	**0.113**	0.057	**0.019**	0.058
I-M3	0.048	0.047	**0.129**	0.057	**0.015**	0.056
I-Y1	0.051	0.049	0.052	0.051	0.056	0.053
I-Y2	0.051	0.049	0.052	0.051	0.056	0.053
I-Y3	0.051	0.049	0.052	0.051	0.056	0.053

*The superscripts “S” and “M” indicate the single-group and multi-group approaches, respectively. W, Wald test; LR, likelihood ratio test. See Table 1 for description of each method. See Table 2 for population parameter values. The Type I error rates that are smaller than 0.025 or greater than 0.075 are shown in bold*.

**Figure 3:**
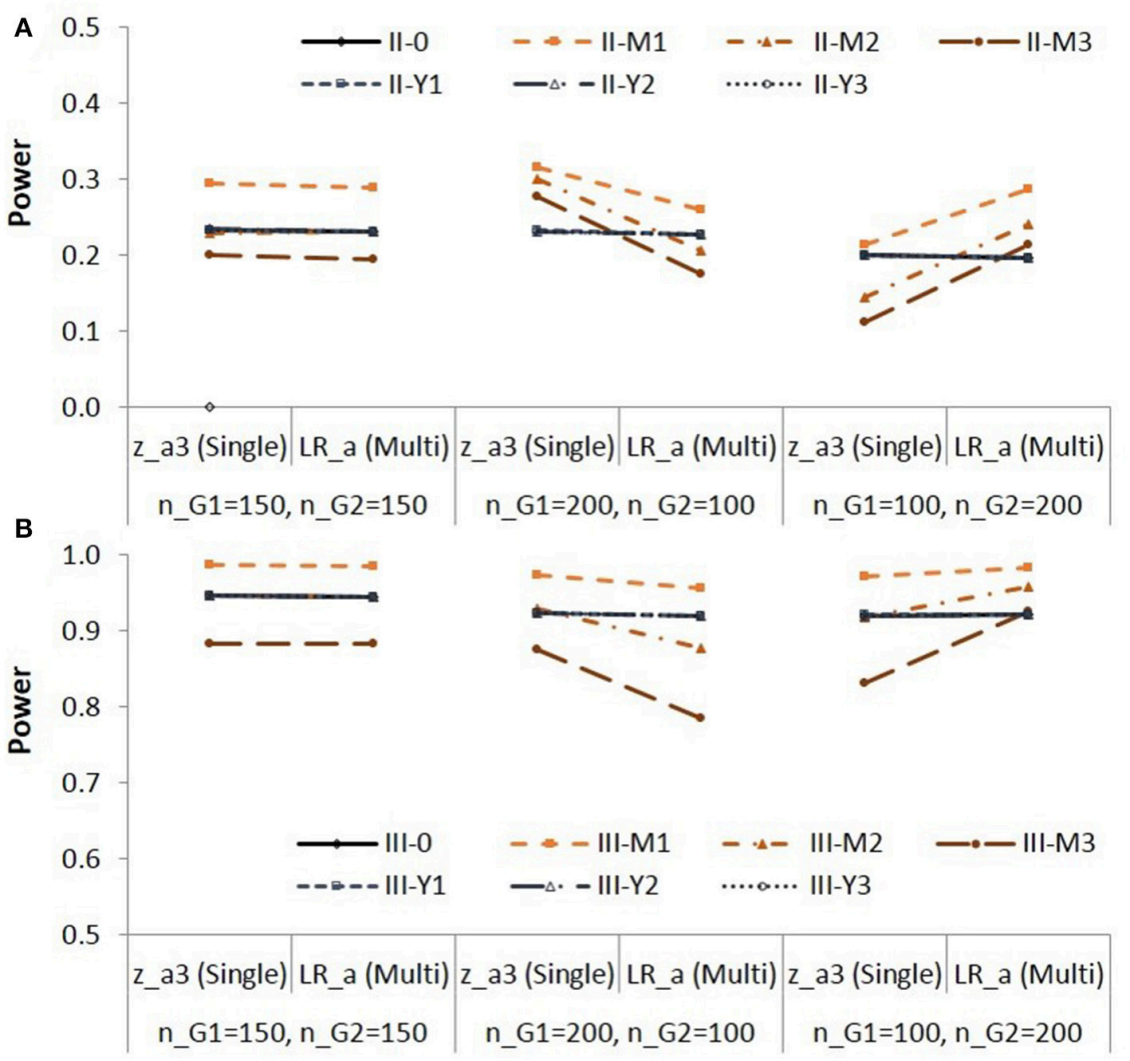
**Empirical power for testing group difference in X to M relationship (*a* path) in Population II (A)** and in Population III **(B)**. See Table 1 for description of the methods.

The Type I error rates of the $LR_{a}^{M}$ method stayed close to the nominal level. But the $z_{a3}^{S}$ method resulted in inflated Type I error rates when the residual variance of M was smaller in the group with a larger sample size (Populations I-M1 to I-M3; *n*_*G*1_ = 200; *n*_*G*2_ = 100). The $z_{a3}^{S}$ method resulted in deflated Type I error rates when the residual variance of M was smaller in the group with a smaller sample size (Populations I-M2 and I-M3; *n*_*G*1_ = 100; *n*_*G*2_ = 200). Whether or not the residual variance of Y was equal between groups did not affect the Type I error rates of the $z_{a3}^{S}$ method. Figure 3 shows the empirical power of the two methods for Populations II and III.

Note that the effect sizes are different in different populations. Figure 3 is to compare the two methods $z_{a3}^{S}$ and $LR_{a}^{M}$ in each condition. When the group sizes were equal, the power was similar for the two methods. When the residual variance of M was not equal (Populations II-M1 to II-M3, Populations III-M1 to III-M3), the $z_{a3}^{S}$ method showed higher power than the $LR_{a}^{M}$ method with the Sample size 2 (*n*_*G*1_ = 200; *n*_*G*2_ = 100); the $z_{a3}^{S}$ method showed lower power than the $LR_{a}^{M}$ method with the Sample size 3 (*n*_*G*1_ = 100; *n*_*G*2_ = 200).

### Group difference in the indirect effect

#### Type I error rates

Table 5 shows the empirical Type I error rates of the methods for testing the group difference in the indirect effect in Population I.

**Table 5:** **Type I error rates of the methods for testing group difference in the indirect effect**.

**Population**	**$W_{diff}^{S}$**	**$LR_{diff}^{M}$**	**$W_{diff}^{M}$**	**$PC_{diff}^{M}$**	**$BC_{diff}^{M}$**	**$MC_{diff}^{M}$**
**SAMPLE SIZE 1: *n*_*G*1_ = 150; *n*_*G*2_ = 150**						
I-0	0.040	0.060	0.058	0.061	0.067	0.062
I-M1	0.037	0.054	0.051	0.049	0.055	0.057
I-M2	0.036	0.057	0.055	0.060	0.062	0.056
I-M3	0.039	0.058	0.053	0.066	0.065	0.063
I-Y1	0.042	0.061	0.062	0.067	0.063	0.060
I-Y2	0.040	0.066	0.065	0.059	0.068	0.065
I-Y3	0.038	0.066	0.062	0.062	0.066	0.065
**SAMPLE SIZE 2: *n*_*G*1_ = 200; *n*_*G*2_ = 100**						
I-0	0.044	0.050	0.054	0.056	0.060	0.051
I-M1	0.063	0.047	0.047	0.058	0.053	0.051
I-M2	**0.086**	0.053	0.055	0.055	0.061	0.053
I-M3	**0.105**	0.057	0.059	0.055	0.062	0.062
I-Y1	0.047	0.056	0.058	0.061	0.064	0.055
I-Y2	0.046	0.058	0.060	0.059	0.064	0.059
I-Y3	0.044	0.057	0.059	0.064	0.070	0.060
**SAMPLE SIZE 3: *n*_*G*1_ = 100; *n*_*G*2_ = 200**						
I-0	0.049	0.054	0.054	0.060	0.059	0.054
I-M1	**0.018**	0.054	0.053	0.054	0.064	0.058
I-M2	**0.011**	0.056	0.056	0.057	0.061	0.060
I-M3	**0.010**	0.055	0.057	0.057	0.058	0.055
I-Y1	0.050	0.058	0.059	0.057	0.064	0.061
I-Y2	0.047	0.060	0.056	0.058	0.064	0.062
I-Y3	0.042	0.060	0.059	0.063	0.065	0.059

*The superscripts “S” and “M” indicate the single-group and multi-group approaches, respectively. W, Wald test; LR, likelihood ratio test; PC, percentile bootstrap; BC, bias-corrected bootstrap; MC, Monte Carlo method. See Table 1 for description of each method. The Type I error rates that are smaller than 0.025 or greater than 0.075 are shown in bold*.

The Type I error rates for the $W_{diff}^{S}$ method were higher than the nominal level when the residual variance of M was smaller in the group with a larger sample size (Populations I-M2 and I-M3; *n*_*G*1_ = 200; *n*_*G*2_ = 100); and the Type I error rates were smaller than the nominal level when the residual variance of M was smaller in the group with a smaller sample size (Populations I-M1 to I-M3; *n*_*G*1_ = 100; *n*_*G*2_ = 200). This is a similar pattern to the Type I error rates of the $z_{a3}^{S}$ method in Table 4.

For the five methods in the multi-group analysis, the Type I error rates ranged from 0.049 to 0.068 with Sample size 1; ranged from 0.047 to 0.070 with Sample size 2; and ranged from 0.053 to 0.065 with Sample size 3. The equality of residual variances of M and Y in the population did not affect the Type I error rates of the five methods in the multi-group analysis. The Type I error rates of the $BC_{diff}^{M}$ method were slightly higher than the Type I error rates of the other methods.

#### Power

**Figure 4:**
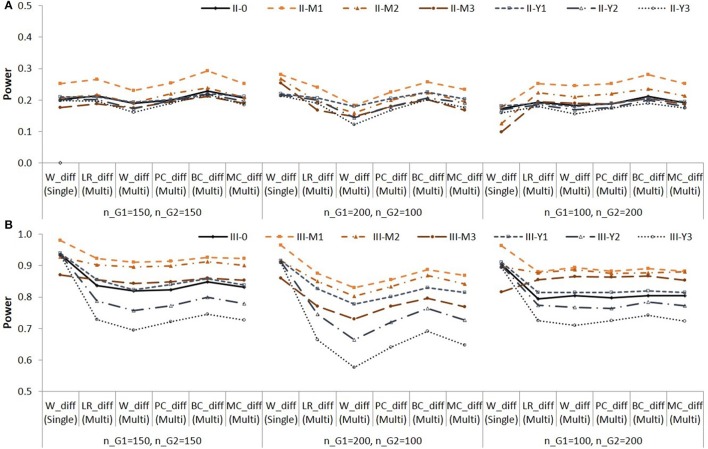
**Empirical power for testing group difference in the indirect effect in Population II (A)** and in Population III **(B)**. See Table 1 for description of the methods.

The empirical power for testing the group difference in the indirect effect in Populations II and III are shown in Figure 4.

Note that the difference in empirical power across populations (i.e., across different lines) are due to different effect sizes as shown in Table 3. The $BC_{diff}^{M}$ method showed higher power than the other methods. The $W_{diff}^{M}$ method showed lower power than the other methods in multi-group analysis. For Population III in which the group difference in the indirect effect was larger, the differences in empirical power between the methods were greater with the sample size *n*_*G*1_ = 200; *n*_*G*2_ = 100, i.e., when the indirect effect was zero in the larger group and larger in the smaller group. When the residual variance of M was not equal between groups (e.g., II-M1,…, II-M3, III-M1,…, III-M3), the $W_{diff}^{S}$ method yielded higher power than the other methods with the sample size *n*_*G*1_ = 200; *n*_*G*2_ = 100. Note that the $W_{diff}^{S}$ method showed inflated Type I error rates in these conditions. The $W_{diff}^{S}$ method yielded lower power than the other methods with the sample size *n*_*G*1_ = 100; *n*_*G*2_ = 200. In these conditions, the Type I error rates were lower than the nominal level.

#### Coverage rates, width, and misses

Three methods in multi-group analysis produced 95% confidence intervals for the group difference in the indirect effect: $PC_{diff}^{M}$, $BC_{diff}^{M}$, and $MC_{diff}^{M}$. The results showed similar patterns in all simulation conditions. The performance of the three confidence intervals was comparable in terms of coverage, width, and misses. The coverage rates of the $PC_{diff}^{M}$ confidence intervals ranged from 0.927 to 0.951 (average = 0.939). The coverage rates of the $BC_{diff}^{M}$ confidence intervals ranged from 0.923 to 0.947 (average = 0.935). The coverage rates of the $MC_{diff}^{M}$ confidence intervals ranged from 0.926 to 0.949 (average = 0.934). On average, the coverage rates were slightly lower than the nominal level. The width of the confidence intervals produced by the three methods was similar to one another. The average width was 0.248 for $PC_{diff}^{M}$, 0.250 for $BC_{diff}^{M}$, and 0.246 for $MC_{diff}^{M}$.

For $PC_{diff}^{M}$, the average ratio of left-to right-side misses was 1.427, 1.927, and 1.824 in Populations I, II, and III, respectively. For $BC_{diff}^{M}$, the average ratio was 1.274, 1.521, and 1.249 in Populations I, II, and III, respectively. For $MC_{diff}^{M}$, the average ratio was 1.397, 1.783, and 1.664 in Populations I, II, and III, respectively. All three confidence intervals showed higher rates of left-side misses than right-side misses^2^. The $BC_{diff}^{M}$ confidence intervals were most balanced (i.e., average ratio closer to 1).

### Simple indirect effect in each group

#### Type I error rates

The indirect effect was zero in Group 1 in Populations II and III. The Type I error rates for testing the simple indirect effect are shown in Table 6. The results were similar in Populations II and III, and the results for Population II are shown in Table 6.

**Table 6:** **Type I error rates for testing simple indirect effect in Group 1 in Population II**.

**Population**	**$PC_{ind}^{S}$**	**$BC_{ind}^{S}$**	**$PC_{ind}^{M}$**	**$BC_{ind}^{M}$**	**$MC_{ind}^{M}$**
**SAMPLE SIZE 1: *n*_*G*1_ = 150; *n*_*G*2_ = 150**					
II-0	0.050	0.065	0.056	0.073	0.049
II-M1	0.048	0.068	0.049	0.075	0.043
II-M2	0.048	0.067	0.047	0.071	0.042
II-M3	0.048	0.066	0.046	0.075	0.040
II-Y1	0.050	0.063	0.053	0.063	0.054
II-Y2	0.050	0.065	0.052	0.064	0.051
II-Y3	0.050	0.067	0.052	0.061	0.046
**SAMPLE SIZE 2: *n*_*G*1_ = 200; *n*_*G*2_ = 100**					
II-0	0.060	0.074	0.062	**0.082**	0.059
II-M1	0.058	0.074	0.061	**0.090**	0.059
II-M2	0.058	0.071	0.062	**0.085**	0.058
II-M3	0.058	0.072	0.055	**0.082**	0.061
II-Y1	0.060	0.066	0.062	0.069	0.061
II-Y2	0.060	0.071	0.063	0.071	0.059
II-Y3	0.060	0.071	0.065	**0.077**	0.061
**SAMPLE SIZE 3: *n*_*G*1_ = 100; *n*_*G*2_ = 200**					
II-0	0.051	0.069	0.053	**0.082**	0.056
II-M1	0.051	0.071	0.039	**0.077**	0.038
II-M2	0.051	0.067	0.040	**0.077**	0.045
II-M3	0.051	0.064	0.042	0.072	0.038
II-Y1	0.051	0.067	0.058	**0.076**	0.059
II-Y2	0.051	0.072	0.061	**0.078**	0.063
II-Y3	0.051	0.072	0.065	**0.077**	0.056

*The superscripts “S” and “M” indicate the single-group and multi-group approaches, respectively. PC, percentile bootstrap; BC, bias-corrected bootstrap; MC, Monte Carlo method. See Table 1 for description of each method. Type I error rates that are smaller than 0.025 or greater than 0.075 are shown in bold*.

In the single-group analysis, the Type I error rates were higher for the $BC_{ind}^{S}$ method than for the $PC_{ind}^{S}$ method. In the multi-group analysis, the $PC_{ind}^{M}$ and $MC_{ind}^{M}$ methods showed the Type I error rates that were close to the nominal level. Overall, the $BC_{ind}^{M}$ method resulted in higher Type I error rates than the $PC_{ind}^{M}$ and $MC_{ind}^{M}$ methods. The Type I error rates of the $BC_{ind}^{M}$ method were greater than 0.075 in some conditions (shown in bold).

#### Power

**Figure 5:**
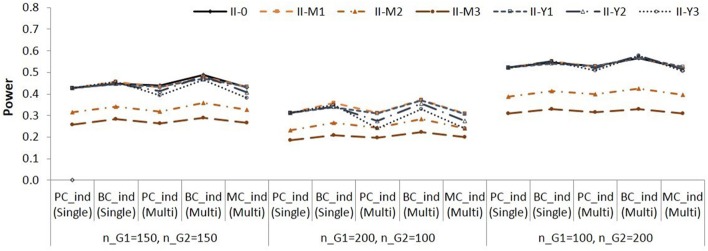
**Empirical power for testing simple indirect effect in Group 2 in Population II**. See Table 1 for description of the methods.

Figure 5 shows the power for testing the simple indirect effect in Group 2 in Population II, in which *a* = 0.141 and *b* = 0.390. When *a* = 0.424 and *b* = 0.390 in population (i.e., both groups in Population I, and Group 2 in Population II), the power for testing the simple indirect effects in each group was very high in all conditions.

Again, note that the difference in empirical power across populations (i.e., across different lines) are due to different effect sizes as shown in Table 3. The $BC_{ind}^{S}$ and $BC_{diff}^{M}$ methods were slightly more powerful than the other methods. The $PC_{ind}^{S}$, $PC_{ind}^{M}$, and $MC_{ind}^{M}$ showed similar power.

#### Coverage rates, width, and misses

**Figure 6:**
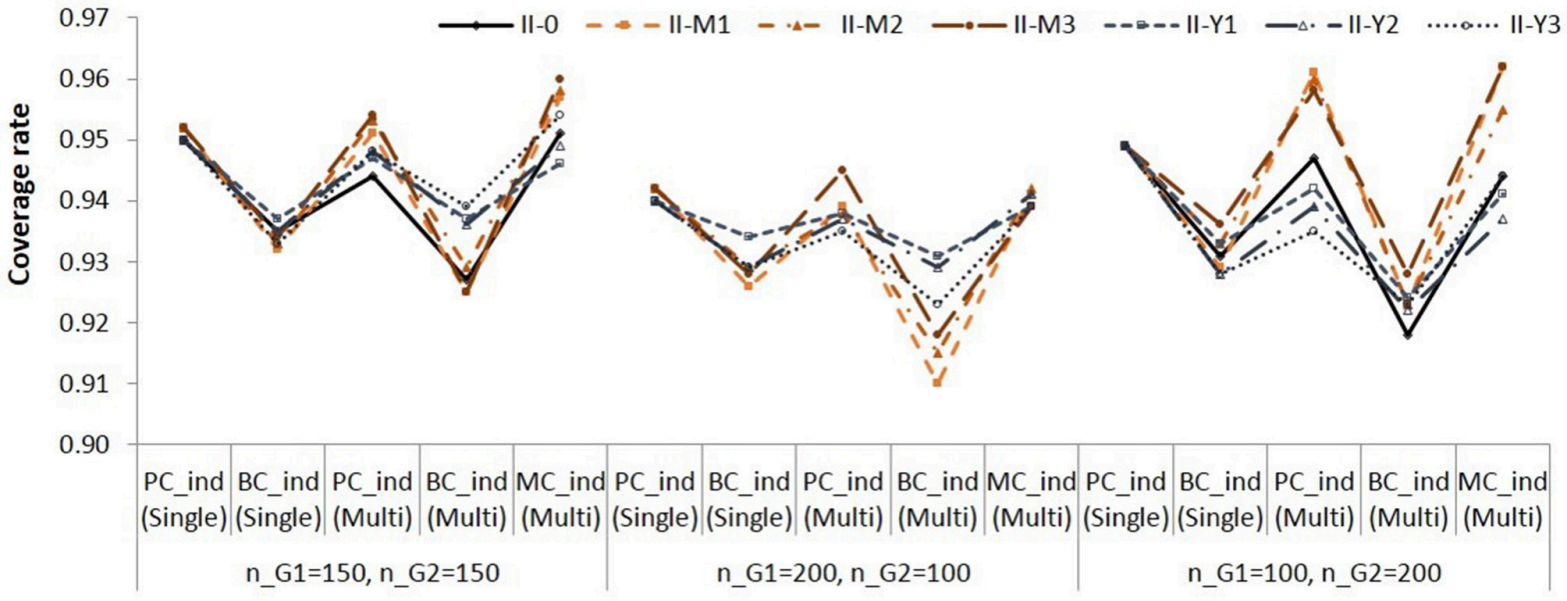
**Coverage rates of 95% confidence intervals for the simple indirect effects in Group 1 in Population II**. See Table 1 for description of the methods.

In the single-group analysis, the coverage rates of the $PC_{ind}^{S}$ confidence intervals ranged from 0.926 to 0.952 (average = 0.939). The coverage rates of the $BC_{ind}^{S}$ confidence intervals ranged from 0.919 to 0.950 (average = 0.934). In the multi-group analysis, the coverage rates ranged from 0.920 to 0.962 (average = 0.937) for the $PC_{ind}^{M}$ method; from 0.910 to 0.953 (average = 0.932) for the $BC_{ind}^{M}$ method; from 0.919 to 0.962 (average = 0.938) for the $MC_{ind}^{M}$ method. The results showed similar pattern in Populations I, II, and III. We present the coverage rates for Group 1 in Population II in Figure 6.

The $BC_{ind}^{S}$ and $BC_{ind}^{M}$ methods yielded lower coverage rates than the other methods. The $PC_{ind}^{S}$, $PC_{ind}^{M}$, and $MC_{ind}^{M}$ methods showed more accurate coverage rates than the $BC_{ind}^{S}$ and $BC_{ind}^{M}$ methods.

On average, the confidence interval methods in the multi-group analysis resulted in wider intervals than those in the single-group analysis. The average width across all conditions was 0.147 for $PC_{ind}^{S}$, and 0.148 for $BC_{ind}^{S}$. In the multi-group analysis, the average width was 0.169 for $PC_{ind}^{M}$, 0.172 for $BC_{ind}^{M}$, and 0.168 for $MC_{ind}^{M}$.

Table 7 shows the average ratio of left- to right-side misses of confidence intervals methods for simple indirect effects.

**Table 7:** **Average ratio of left-to-right misses of confidence intervals methods for simple indirect effects**.

	**Population I**		**Population II**		**Population III**	
	**Group1^a^**	**Group2^a^**	**Group1^b^**	**Group2^a^**	**Group1^b^**	**Group2^a^**
$PC_{ind}^{S}$	0.627	0.486	1.674	0.606	1.674	0.494
$BC_{ind}^{S}$	0.969	0.791	1.467	0.770	1.476	0.790
$PC_{ind}^{M}$	0.613	0.474	1.670	0.493	1.644	0.491
$BC_{ind}^{M}$	1.025	0.851	1.491	0.660	1.486	0.886
$MC_{ind}^{M}$	0.604	0.506	1.626	0.488	1.638	0.499

*The superscripts “S” and “M” indicate the single-group and multi-group approaches, respectively. PC, percentile bootstrap; BC, bias-corrected bootstrap; MC, Monte Carlo method. See Table 1 for description of each method*.

a *The simple indirect effect was positive in population*.

b *The simple indirect effect was zero in population*.

The confidence intervals showed higher rates of right-side misses for the simple indirect effects whose population values were positive, except $BC_{ind}^{M}$ in Population I. The confidence intervals showed higher rates of left-side misses for simple indirect effects whose population values were zero. Both in the single-group and multi-group analysis, the bias-corrected confidence intervals, $BC_{ind}^{S}$ and $BC_{ind}^{M}$, were most balanced (i.e., average ratio closer to 1).

## Empirical example

**Figure 7:**
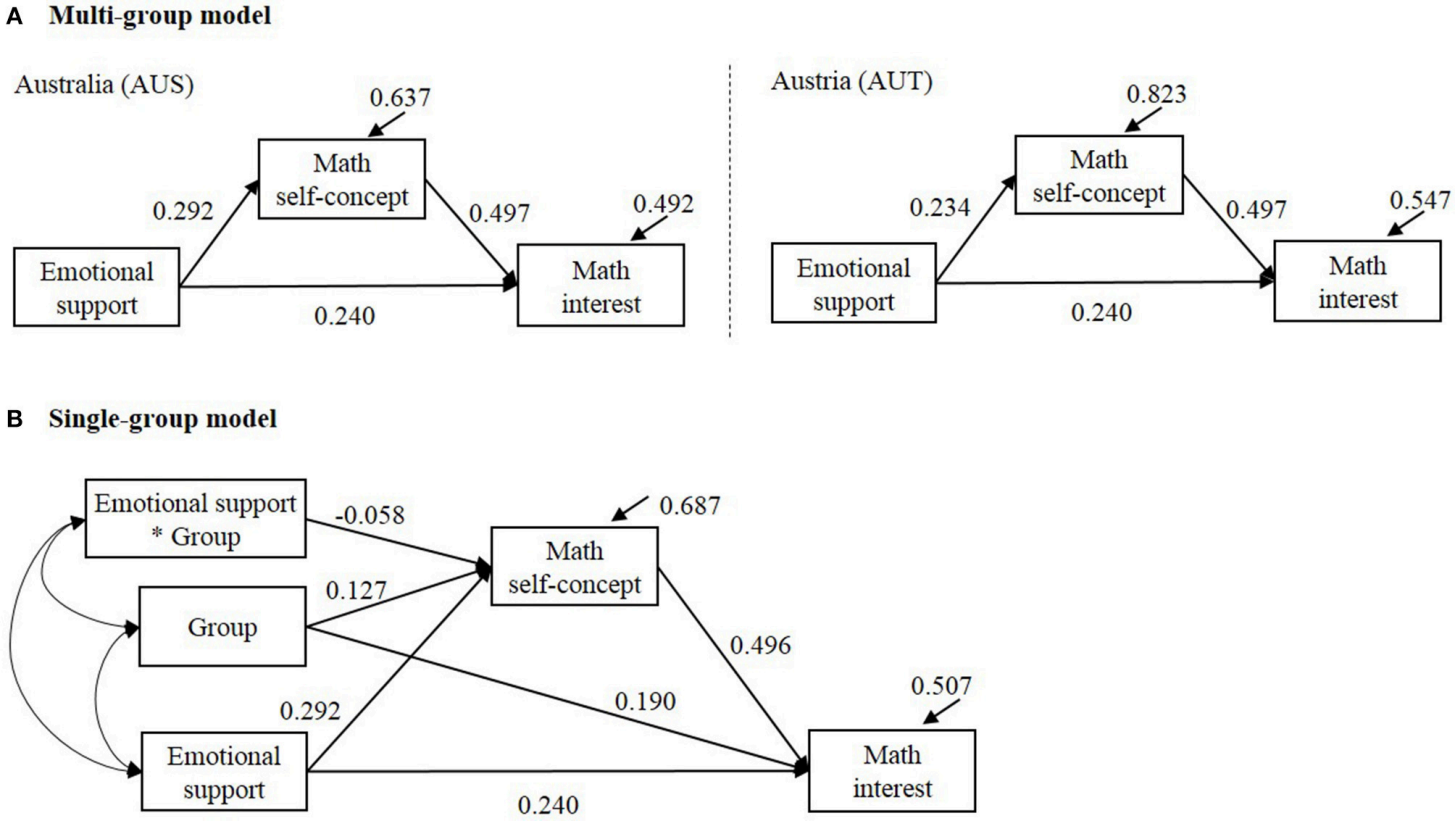
**Estimated multi-group and single-level structural equation models**. In **(A)** Multi-group model, the path coefficient “Math self-concept → Math interest” was set equal between groups; the path coefficient “Emotional support → Math interest” was set equal between groups. The estimate of the indirect effect was 0.292^*^0.497 = 0.145 in Australia (AUS) and 0.234^*^0.497 = 0.116 in Austria (AUT). In **(B)** single-group model, group was coded 0 = AUS and 1 = AUT. The estimated indirect effect was 0.292^*^0.496 = 0.145 for AUS and (0.292–0.058)^*^0.496 = 0.116 for AUT. The estimated group difference in the indirect effect was 0.116–0.145 = –0.029.

We illustrate the methods using empirical data from PISA 2003 database (Programme for International Student Assessment, Organisation for Economic Co-operation Development, 2004, 2005). We adopted a conceptual model in Yeung (2007). We compared the indirect effect of teachers' emotional support on math interest via math self-concept in Australia (AUS; *N* = 1,2551) and Austria (AUT; *N* = 4,597). The estimated multi-group and single-group structural equation models are shown in Figure 7. We applied the methods for (i) comparing the *a* path between groups, (ii) comparing the indirect effect between groups, (iii) testing simple indirect effect in each group. In the multi-group model (Figure 7A), with the *b* path (Math self-concept → Math interest) and *c*′ path (Emotional support → Math interest) set equal between groups, χ^2^(2) = 0.464, *p* = 0.793, CFI = 1.000, RMESA = 0.000, SRMR = 0.003. We kept the equality constrains on *b* and *c*′ paths in the multi-group model so that the specification of the fixed effects is equivalent to the single-group model. In the single-group model (Figure 7B), we created a group variable to represent the two countries that 0 = Australia (AUS) and 1 = Austria (AUT). The results are summarized in Table 8. In the multi-group model, the residual variances were slightly smaller in AUS whose sample size was larger. This is similar to Sample size 2 (*n*_*G*1_ = 200; *n*_*G*2_ = 100) condition in the simulation. In Table 8, $LR_{a}^{M}$ was slightly more conservative than $z_{a3}^{S}$ in testing the group difference in *a* path; $LR_{diff}^{M}$ and $W_{diff}^{M}$ were slightly more conservative than $W_{diff}^{S}$ in testing the group difference in the indirect effect. For the difference in the indirect effect, $PC_{diff}^{M}$, $BC_{diff}^{M}$, and $MC_{diff}^{M}$ yielded in comparable results. For the simple indirect effect, $PC_{ind}^{S}$, $BC_{ind}^{S}$, $PC_{ind}^{M}$, $BC_{ind}^{M}$, and $MC_{ind}^{M}$ resulted in comparable interval estimates.

**Table 8:** **Empirical example results**.

**Method**	**Result**
**GROUP DIFFERENCE IN *a* PATH**	
$z_{a3}^{S}$	â_3_ = −0.058, standard error = 0.020, *p* = 0.005
$LR_{a}^{M}$	LR statistic = 7.122, df = 1, *p* = 0.0076
**GROUP DIFFERENCE IN THE INDIRECT EFFECT**	
$W_{diff}^{S}$	Wald statistic = 7.903, df = 1, *p* = 0.0049
$LR_{diff}^{M}$	LR statistic = LR statistic = 7.122, df = 1, *p* = 0.0076
$W_{diff}^{M}$	Wald statistic = 7.115, df = 1, *p* = 0.0076
$PC_{diff}^{M}$	95% confidence intervals = (–0.057, –0.001)
$BC_{diff}^{M}$	95% confidence intervals = (–0.057, –0.001)
$MC_{diff}^{M}$	95% confidence intervals = (–0.051, –0.008)
**SIMPLE INDIRECT EFFECT IN EACH GROUP**	
$PC_{ind}^{S}$	95% confidence intervals = (0.128, 0.161) in AUS; (0.093, 0.139) in AUT
$BC_{ind}^{S}$	95% confidence intervals = (0.129, 0.161) in AUS; (0.093, 0.140) in AUT
$PC_{ind}^{M}$	95% confidence intervals = (0.128, 0.162) in AUS; (0.093, 0.139) in AUT
$BC_{ind}^{M}$	95% confidence intervals = (0.130, 0.163) in AUS; (0.092, 0.139) in AUT
$MC_{ind}^{M}$	95% confidence intervals = (0.134, 0.157) in AUS; (0.098, 0.135) in AUT

*See Table 1 for description of each method*.

## Summary and discussion

When the research question involves comparing indirect effects between distinctive groups, researchers can choose single-group or multi-group analysis approach in SEM framework to incorporating the group membership as a categorical moderator. In this article, we evaluated statistical methods for (i) comparing a structural path (in our example, *a* path or X → M relationship) between groups, (ii) comparing the indirect effect between groups, and (iii) testing simple indirect effect in each group. We continue to use the abbreviated names of each method to summarize and discuss the results (See Table 1).

The key findings in the simulation study are:

1. In the single-group analysis, the $z_{a3}^{S}$ and $W_{diff}^{S}$ methods may result in invalid statistical inferences when the assumption of equal variances is neglected.
2. However, the performance of bootstrapping confidence intervals is robust even when the bootstrap estimates are obtained in the single-group model.
3. The bias-corrected bootstrap confidence intervals are slightly more powerful than the percentile bootstrap and Monte Carlo confidence intervals, but at the cost of higher Type I error rate, and;
4. For comparing an indirect effect between groups, the likelihood ratio test in the multi-group analysis is as powerful as the other methods with the Type I error rate staying close to the desired level.

For testing the group difference in the *a* path, the assumption of equal variances was critical for the $z_{a3}^{S}$ method, but not for the $LR_{a}^{M}$ method in the multi-group analysis. When the assumption was not satisfied, the $z_{a3}^{S}$ method showed inaccurate Type I error rates, as expected. The Type I error rates were inflated when the variance was larger in the smaller group, and deflated when the variance was larger in the larger group.

For testing the simple indirect effect in each group, the bootstrap confidence intervals in the single-group analysis ($PC_{ind}^{S}$, $BC_{ind}^{S}$) were not affected by the violation of the equal variances assumption. The $PC_{ind}^{S}$ and $BC_{ind}^{S}$ confidence intervals were obtained based on the set of 1,000 estimates in bootstrap samples. As shown in the simulation results, the estimates in the single-group analysis model were unbiased regardless of whether the assumption of equal variances is satisfied. So the empirical sampling distribution of the indirect effect is expected to be comparable with or without the assumption of equal variances satisfied. Therefore, the bootstrap confidence intervals obtained from the empirical sampling distribution were not affected by the assumption.

In the multi-group analysis, all methods did not show differences in their performance depending on whether or not the equal variances assumption is satisfied. These results were expected, because the variances were estimated in each group separately in the multi-group model.

In both single-group and multi-group approaches, the bias-corrected bootstrap methods ($BC_{ind}^{S}$, $BC_{ind}^{M}$, $BC_{diff}^{M}$) tended to show slightly higher Type I error rates, higher statistical power, and lower coverage rates than the percentile bootstrap methods ($PC_{ind}^{S}$, $PC_{ind}^{M}$, $PC_{diff}^{M}$). This pattern of results is consistent with what has been found in previous studies (e.g., Preacher et al., 2007; Preacher and Selig, 2012; Ryu, 2015). The Monte Carlo methods ($MC_{ind}^{M}$, $MC_{diff}^{M}$) performed similarly to the percentile bootstrap methods. The Type I error rates and the coverage rates of the confidence intervals were close to the desired level in all conditions. The empirical power was slightly lower than the bias-corrected bootstrap methods, but not by much. The largest difference in power was 0.091.

For the interval estimates of the group difference in the indirect effect, the average widths were comparable for all three methods in the multi-group analysis ($PC_{diff}^{M}$, $BC_{diff}^{M}$, $MC_{diff}^{M}$). For the interval estimates of the simple indirect effects, the two methods in the single-group analysis ($PC_{ind}^{S}$, $BC_{ind}^{S}$) showed similar average widths, and the three methods in the multi-group analysis ($PC_{ind}^{M}$, $BC_{ind}^{M}$, $MC_{ind}^{M}$) showed similar average widths. The multi-group methods resulted in wider interval estimates of the simple indirect effects than the single-group methods.

The confidence intervals for the simple indirect effects were unbalanced with higher rate of left-side misses when the simple indirect effect was zero in population, and unbalanced with higher rate of right-side misses when there was a positive simple indirect effect in population. For both the group difference in the indirect effect and the simple indirect effects, the bias-corrected bootstrapping methods ($BC_{diff}^{M}$, $BC_{ind}^{S}$, $BC_{ind}^{M}$) were most balanced in terms of the ratio of left- and right-side misses.

In the multi-group analysis, the likelihood ratio test ($LR_{diff}^{M}$) and the Wald test ($W_{diff}^{M}$) performed well in terms of Type I error rates. But the $W_{diff}^{M}$ method showed lower power than the $LR_{diff}^{M}$ and the confidence intervals methods for testing the group difference in the indirect effect. The empirical power of the $LR_{diff}^{M}$ method was comparable to the power of $PC_{diff}^{M}$ and $MC_{diff}^{M}$. These results are consistent with those found in a previous study (Ryu, 2015). In the single-group analysis, the performance of the Wald test ($W_{diff}^{S}$) for testing the group difference in the indirect effect was affected by the violation of the equal variance assumption, particularly with unequal group sizes. The Type I error rates were higher than the desired level when the variance was larger in the smaller group. The Type I error rates were smaller than the nominal level when the variance was larger in the larger group.

In many cases, studies are conducted to address questions on means (unconditional or conditional) and relationships between variables, and the variance estimates are often neglected. It is important for researchers to pay attention to variance estimates, even when they are not of key interest. When the research question involves moderation effect by a distinctive group membership, it is recommended that the variance parameters are examined first with no restriction that the variances are equal in all groups. When it is reasonable to assume that the variances are equal, researchers may choose to adopt single-group or multi-group analysis approach. When it is not reasonable to assume equal variances, multi-group analysis is recommended. The single-group analysis resulted in unbiased parameter estimates even with the assumption violated. But some methods for statistical inference were affected by the violation of the assumption. If single-group analysis is adopted, statistical methods must be chosen with careful consideration.

Multi-group analysis approach has advantages over single-group approach in incorporating a categorical moderator in the model. First, the multi-group approach does not depend on the assumption of equal variances, and so the parameter estimates and statistical inferences are not affected by the assumption satisfied or violated. Second, it is less complicated to specify and test the group difference in more than one indirect effect. For example, suppose that a mediation model is hypothesized in which three indirect effects are specified between one independent variable (X), three mediating variables (M1, M2, and M3), and one dependent variable (Y). In order to specify a model that allows the three indirect effects to differ between groups, the single-group approach requires at least three additional product terms to represent the interaction with the group membership. The number of required product terms can increase if there are more than two levels of the categorical moderator, or if both the relationship between X and the mediators and the relationship between the mediators and Y differ between groups. In the multi-group analysis, however, the group differences can be specified and tested without increasing the number of variables in the model.

In conclusion, when the data are from more than one distinctive group, we recommend that researchers first examine parameter estimates (including variance parameters) in each group with no restriction before choosing to adopt single-group analysis. For testing the group difference in the indirect effect in multi-group analysis, the likelihood ratio test is more powerful than Wald test, with Type I error rate close to the desired level. For confidence intervals of the group difference in the indirect effect, bias-corrected bootstrap confidence intervals were more powerful and more balanced than the percentile bootstrap and Monte Carlo confidence intervals, but at the cost of higher Type I error rates and lower coverage rates. For the simple indirect effect in each group, bias-corrected bootstrap confidence intervals were more powerful than the percentile bootstrap and Monte Carlo confidence intervals, but again the Type I error rates were higher with bias-corrected bootstrap confidence intervals. Taken together, we recommend the likelihood ratio test along with the percentile or Monte Carlo interval estimates for the group difference in the indirect effect. We recommend the percentile or Monte Carlo interval estimates for the simple indirect effect.

## Author contributions

ER and JC designed the study. ER conducted the simulation study and took a leading role in writing the manuscript. JC conducted a part of the simulation and participated in writing.

### Conflict of interest statement

The authors declare that the research was conducted in the absence of any commercial or financial relationships that could be construed as a potential conflict of interest.

## Footnotes

^1^In mediation analysis, the poor performance of the method based on the normality assumption is well-known. We included the normal theory standard error method in the simulation study. As expected, and consistent with the previous findings in the literature, the normal standard error method did not perform well. We introduce the method here for the purpose of reviewing previous literature but do not consider the normal-theory standard error method hereafter to avoid redundancy. The normal-theory standard error does not appear in Table 1. We do not present simulation results regarding this method.

^2^The confidence intervals were obtained for (*a*_*G*1_*b*_*G*1_−*a*_*G*2_*b*_*G*2_). The rates of left-side and right-side misses would be reversed if the group difference is calculated in the opposite direction.
