# Supplementary material for: Comparing Indirect Effects in Different Groups in Single-Group and Multi-Group Structural Equation Models
Source: Front Psychol. 2017 May 11;8:747. doi: 10.3389/fpsyg.2017.00747 (PMC5425601; doi:10.3389/fpsyg.2017.00747)
Supplement: Supplementary file 1 [file DataSheet1.docx]

**Appendix**

SAS macro to generate bootstrap samples from multi-group data with 2 groups

/***************************************************************************/

/** SAS macro to generate bootstrap samples from multi-group data **/

/** with 2 groups **/

/** The generated bootstrap samples will have the same group sizes **/

/** as those in the original sample **/

/** The bootstrap sample data will be saved bt1.dat, bt2.dat, etc. **/

/** The code recognizes the original sample data as working data “a” **/

/***************************************************************************/

**%macro** ***boot***;

/* The following input must be provided by the user */

%let nboot=; /*number of bootstrap samples (e.g., 1000)*/

%let ng1=; /*group 1 sample size (e.g., 12551)*/

%let ng2=; /*group 2 sample size (e.g., 4597)*/

%let group=; /*name of group variable (e.g., country)*/

%let g1=; /*coding of group 1 (e.g., 036)*/

%let g2=; /*coding of group 2 (e.g., 040)*/

%let path=; /*location of a folder in which the bootstrap samples to be saved (e.g., C:\folder)*/

%let format=; /*format to save bootstrap sample data into ascii file (. dat) (e.g., @1 country 40.0 @5 x 8.4 @13 m 8.4 @21 y 8.4)*/

/* No need to alter the code below */

data g1; set a; if &group=&g1; data g2; set a; if &group=&g2;

%do boot=**1** %to &nboot;

data boot_g1;

%do obs=**1** %to &ng1;

rs=round(ranuni(**0**)*&ng1);

if rs=**0** then rs=**1**; set g1 point=rs;

if _error_=**1** then stop;

output;

%end;

stop;

data boot_g2;

%do obs=**1** %to &ng2;

rs=round(ranuni(**0**)*&ng2);

if rs=**0** then rs=**1**; set g2 point=rs;

if _error_=**1** then stop;

output;

%end;

stop;

data boot; set boot_g1 boot_g2;

data _null_; set boot;

file "&path\bt&boot..dat"; put &format;

%end;

**%mend**;

%***boot***;

**run**; **quit**;

SAS macro to find percentile and bias-corrected confidence interval from a set of bootstrap estimates

/***************************************************************************/

/** SAS macro to find percentile and bias-corrected bootstrap confidence **/

/** interval from a set of bootstrap estimate **/

/** The code recognizes the data that contains the bootstrap estimates **/

/** as working data "bootest" **/

/***************************************************************************/

**%macro** ***bootci***;

/* The following input must be provided by the user */

%let nboot=; /*number of bootstrap samples (e.g., 1000)*/

%let sampest=; /*the estimate in the original sample (e.g., -0.028826)*/

%let btestname=; /*name of the variable that contains the bootstrap estimate (e.g., inddiff)*/

%let clpct=; /*level of confidence (e.g., 95)*/

/* No need to alter the code below */

data samp; est=&sampest; mergeid=**1**;

data boot; set bootest; btest=&btestname; mergeid=**1**;

data estbtest; merge samp boot; by mergeid;

data _NULL_;

lowerpct=(**100**-&clpct)/**2**;

upperpct=**100**-lowerpct;

call symput('lowerpct',lowerpct);

call symput('upperpct',upperpct);

data pctboot; set _null_;

proc univariate data=estbtest noprint; var &btestname;

output out=pctboot n=nbt pctlpts=&lowerpct &upperpct pctlpre=&btestname._ pctlname=lcl ucl;

data pctboot2; set pctboot;

data findp; set estbtest; if &btestname > &sampest then p=**1**; else p=**0**;

proc means data=findp noprint; output out=findp2 mean(p)=p;

data _NULL_; set findp2; z0=probit(**1**-p); call symput('z0',z0);

data _NULL_;

zp=probit(&upperpct/**100**);

roundpoint=**100**/&nboot;

bcbzlo=(**2***&z0)-zp; bcbzup=(**2***&z0)+zp;

bcbplo=probnorm(bcbzlo); bcbpup=probnorm(bcbzup);

bcbpctlo=round(bcbplo***100**,roundpoint);

bcbpctup=round(bcbpup***100**,roundpoint);

call symput('bcbpctlo',bcbpctlo);

call symput('bcbpctup',bcbpctup);

data bcboot; set _null_;

proc univariate data=estbtest noprint; var &btestname;

output out=bcboot pctlpts=&bcbpctlo &bcbpctup pctlpre=bcb_&btestname._ pctlname=lcl ucl;

data bcboot2; set bcboot;

data bootci; merge pctboot2 bcboot2; proc print data=bootci;

**%mend**;

%***bootci***;

**run**; **quit**;

SAS macro to generate Monte Carlo confidence intervals for the group difference in the indirect effect and for the simple indirect effect in each group

/***************************************************************************/

/** SAS macro to find Monte Carlo confidence interval **/

/** for the simple indirect effect in each group ("indg1", "indg2") and **/

/** for the group difference in the indirect effect ("inddiff") **/

/** inddiff = indg2 - indg1 **/

/** The user must provide the estimates and standard error of **/

/** a and b paths in each group **/

/***************************************************************************/

**%macro** ***mc***;

%let mcrep=1000; /*number of Monte Carlo replications (e.g., 1000)*/

%let a1=0.292; /*estimate of a path in group 1 (e.g., 0.292)*/

%let ase1=0.011; /*standard error of a path in group 1 (e.g., 0.011)*/

%let b1=0.497; /*estimate of b path in group 1 (e.g., 0.497)*/

%let bse1=0.007; /*standard error of b path in group 1 (e.g., 0.007)*/

%let a2=0.234; /*estimate of a path in group 2 (e.g., 0.234)*/

%let ase2=0.018; /*standard error of a path in group 2 (e.g., 0.018)*/

%let b2=0.497; /*estimate of b path in group 2 (e.g., 0.497)*/

%let bse2=0.007; /*standard error of b path in group 2 (e.g., 0.007)*/

%let clpct=95; /*level of confidence (e.g., 95)*/

data est;

a_g1=&a1; ase_g1=&ase1; b_g1=&b1; bse_g1=&bse1;

a_g2=&a2; ase_g2=&ase2; b_g2=&b2; bse_g2=&bse2;

proc iml;

use est; read all var {a_g1} into a_g1;

use est; read all var {a_g2} into a_g2;

use est; read all var {b_g1} into b_g1;

use est; read all var {b_g2} into b_g2;

use est; read all var {ase_g1} into ase_g1;

use est; read all var {ase_g2} into ase_g2;

use est; read all var {bse_g1} into bse_g1;

use est; read all var {bse_g2} into bse_g2;

avar_g1=ase_g1****2**; avar_g2=ase_g2****2**;

bvar_g1=bse_g1****2**; bvar_g2=bse_g2****2**;

g1est=a_g1//b_g1; g2est=a_g2//b_g2;

g1cov=(avar_g1||**0**)//(**0**||bvar_g1); g2cov=(avar_g2||**0**)//(**0**||bvar_g2);

do i=**1** to &mcrep;

call vnormal (g1mc,g1est,g1cov,**1**);

a_g1=g1mc[,**1**]; b_g1=g1mc[,**2**];

call vnormal (g2mc,g2est,g2cov,**1**);

a_g2=g2mc[,**1**]; b_g2=g2mc[,**2**];

indg1=a_g1*b_g1;

indg2=a_g2*b_g2;

inddiff=indg2-indg1;

mc=i||indg1||indg2||inddiff;

mcall=mcall//mc;

end;

colname={"mc" "indg1" "indg2" "inddiff"};

create mcall from mcall [colname=colname];

append from mcall;

data _NULL_;

lowerpct=(**100**-&clpct)/**2**;

upperpct=**100**-lowerpct;

call symput('lowerpct',lowerpct);

call symput('upperpct',upperpct);

data mcindg1; set _null_;

proc univariate data=mcall noprint; var indg1;

output out=mcciindg1 pctlpts=&lowerpct &upperpct pctlpre=indg1_ pctlname=lcl ucl;

proc print data=mcciindg1;

data mcindg2; set _null_;

proc univariate data=mcall noprint; var indg2;

output out=mcciindg2 pctlpts=&lowerpct &upperpct pctlpre=indg2_ pctlname=lcl ucl;

proc print data=mcciindg2;

data mcinddiff; set _null_;

proc univariate data=mcall noprint; var inddiff;

output out=mcciinddiff pctlpts=&lowerpct &upperpct pctlpre=inddiff_ pctlname=lcl ucl;

proc print data=mcciinddiff;

**%mend**;

%***mc***;

**run**; **quit**;

Mplus syntax: Wald test for testing $a_{3}b=0$ in single-group analysis

Data:

file is example.dat;

Variable:

names are group x m y; !group must be appropriately coded;

usevariables are group x m y xgroup;

Define:

xgroup=x*group;

Model:

m on x group;

m on xgroup (a3);

y on x;

y on m (b);

Model test:

0=(a3*b);

Mplus syntax: Likelihood ratio test for testing $a_{G1}b_{G1}=a_{G2}b_{G2}$ in multi-group analysis

!Likelihood ratio test must be computed between the models with and without the constraint (a_G1)*(b_G1) = (a_G2)*(b_G2);

Data:

file is example.dat;

Variable:

names are group x m y;

usevariables are group x m y xgroup;

grouping is group (0=g1 1=g2);

Model:

m on x;

y on x m;

Model g1:

m on x (a1);

y on m (b1);

Model g2:

m on x (a2);

y on m (b2);

Model constraint:

0=(a1*b1)-(a2*b2);

Mplus syntax: Wald test for testing $a_{G1}b_{G1}=a_{G2}b_{G2}$ in multi-group analysis

Data:

file is example.dat;

Variable:

names are group x m y;

usevariables are group x m y xgroup;

grouping is group (0=g1 1=g2);

Model:

m on x;

y on x m;

Model g1:

m on x (a1);

y on m (b1);

Model g2:

m on x (a2);

y on m (b2);

Model test:

0=(a1*b1)-(a2*b2);

Mplus syntax for data generation (Population I-0)

Montecarlo:

names=x m y;

nobs=150 150;

ngroup=2;

nreps=1000;

repsave=all;

save=rep*.dat;

Model population:

m on x*0.424;

y on x*0 m*0.39;

Model population-g1:

m on x*0.424;

y on x*0 m*0.39;

x*1 m*1 y*1;

Model population-g2:

m on x*0.424;

y on x*0 m*0.39;

x*1 m*1 y*1;
